# Perspectives on the Role of Magnetic Resonance Imaging (MRI) for Noninvasive Evaluation of Diabetic Kidney Disease

**DOI:** 10.3390/jcm10112461

**Published:** 2021-06-02

**Authors:** José María Mora-Gutiérrez, María A. Fernández-Seara, Rebeca Echeverria-Chasco, Nuria Garcia-Fernandez

**Affiliations:** 1 Nephrology Department, Clínica Universidad de Navarra, 31008 Pamplona, Spain; jmora@unav.es (J.M.M.-G.); nrgarcia@unav.es (N.G.-F.); 2 Radiology Department, Clínica Universidad de Navarra, 31008 Pamplona, Spain; recheverriac@unav.es; 3 IdiSNA, Navarra Institute for Health Research, 31008 Pamplona, Spain; 4 Red de Investigación Renal (REDINREN), Instituto de Salud Carlos III, 28029 Madrid, Spain

**Keywords:** diabetic kidney disease, magnetic resonance imaging, arterial spin labeling, blood oxygen-dependent level, diffusion weighted imaging, oxygenation, perfusion, fibrosis, chronic kidney disease, kidney function

## Abstract

Renal magnetic resonance imaging (MRI) techniques are currently in vogue, as they provide in vivo information on renal volume, function, metabolism, perfusion, oxygenation, and microstructural alterations, without the need for exogenous contrast media. New imaging biomarkers can be identified using these tools, which represent a major advance in the understanding and study of the different pathologies affecting the kidney. Diabetic kidney disease (DKD) is one of the most important diseases worldwide due to its high prevalence and impact on public health. However, its multifactorial etiology poses a challenge for both basic and clinical research. Therefore, the use of novel renal MRI techniques is an attractive step forward in the comprehension of DKD, both in its pathogenesis and in its detection and surveillance in the clinical practice. This review article outlines the most promising MRI techniques in the study of DKD, with the purpose of stimulating their clinical translation as possible tools for the diagnosis, follow-up, and monitoring of the clinical impacts of new DKD treatments.

## 1. Introduction

Diabetes mellitus (DM) is one of the most prevalent chronic diseases worldwide, and its incidence and prevalence continue to increase [1]. Diabetic kidney disease (DKD) is a major long-term complication of DM and the leading cause of kidney failure [2,3]. Microcirculatory disturbances have been recognized as an early and consistent pathogenic trigger of DKD, although different clinical phenotypes and mechanisms related to the progression of DKD have been identified. Currently, diagnostic and predictive tests available in clinical practice are not yet adequate to detect early DKD and predict the different patterns of DKD progression [4]. The use of renal magnetic resonance imaging (MRI) techniques is breaking new ground in the microstructural evaluation of the kidney [5], providing a better understanding of the pathophysiology at different phases of DKD. In addition, these tools are expected to facilitate the early detection of DKD, as well as its progression phenotypes, inform the decision-making/monitoring process for therapies, and pave the way to a more personalized medical care.

This review paper outlines the most promising MRI techniques in the study of DKD, with the purpose of stimulating their clinical investigation as possible tools for the diagnosis, follow-up, and monitoring of the clinical impacts of new DKD treatments.

## 2. Pathogenesis and Natural History of DKD

Diabetic kidney disease is a condition caused by a dynamic process involving multiple self-reinforcing mechanisms. The paradigm of its natural history continues to evolve. In the past, alterations were described in diverse renal compartments, which caused heterogeneous clinical features. The main clinical manifestation is the presence of albuminuria, which can range from mildly increased albuminuria (albumin–creatinine ratio (ACR) > 30 mg/g) to moderate albuminuria (ACR 30–300 mg/g) or severe albuminuria (ACR > 300 mg/g). Nevertheless, the absence of albuminuria does not exclude the presence of DKD [6]. A prospective follow-up [7] of type 1 diabetes (T1D) patients over 25 years showed that the development of severe albuminuria was rare before 10 years of diabetes duration and increased steadily thereafter. However, after the diagnosis of severe albuminuria, it was observed a 52% cumulative incidence of regression of albuminuria to less than 300 mg/g at 10 years and a 13% cumulative incidence of regression to less than 30 mg/g. Another study [8] followed T1D subjects for an average of 12 years, after the onset of mildly increased albuminuria, and observed that almost one-third of the patients progressed to kidney failure, although nearly half had no significant variations in albuminuria levels. A previous work [9] analyzing the 10 year evolution of kidney function in 4041 type 2 diabetes (T2D) patients, showed that 28% of patients presented no decline in the glomerular filtration rate (GFR), 56% had a moderate decline, and 15% developed a severe decline.

In DKD, the urinary sediment is usually bland; however, the presence of microscopic hematuria may occur as in any form of glomerular disease [10]. This is important since nondiabetic kidney disease is occasionally seen in diabetic patients, either alone or with superimposed DKD [11]. Depending on the population studied, the most frequent glomerulopathy may be focal segmental glomerulosclerosis (FSGS) [12], hypertensive nephrosclerosis [13], membranous nephropathy, or IgA nephropathy [14].

In contrast to these features, an autopsy study (*n* = 168) revealed a DKD prevalence of 63% in diabetic subjects with a history of T1D and T2D. Interestingly, 19% of the subjects had never experienced clinical manifestations of DKD during their lifetime. Among these underdiagnosed subjects, a variety of diverse histologic patterns were found. These findings suggest that DKD lesions precede the appearance of clinical manifestations [3].

The classic histological features of DKD include loss of endothelial fenestrations, thickening of capillary, tubular, and glomerular basement membranes, expansion of the mesangial matrix, and loss of podocytes [15]. Subsequently, segmental mesangiolysis, Kimmelstiel–Wilson nodules, and microaneurysms are observed [16].

It is now recognized that endothelial and capillary disruption play an early and consistent role in the development of DKD by compromising the balance of oxygen supply/consumption in the tissues. Therefore, hypoxia is recognized as an important factor in the development and progression of the disease. Primary microcirculation impairment leads to reduced renal blood flow (RBF), which decreases oxygen supply and generates segmental hypoxic lesions. Subsequently, secondary hyperfiltration by unaffected glomeruli results in increased oxygen consumption and exacerbation of hypoxic renal injury [17]. Furthermore, two of the main risk factors for progression of DKD (hyperglycemia and hypertension) are implicated in the compromise of renal oxygen supply by promoting ATP production/demand imbalance [18]. The Na^+^/K^+^-ATPase transporter consumes the greatest amount of renal oxygen (ATP consumption) during tubular sodium reabsorption. In addition, increased glucose reabsorption in the proximal tubule of diabetic patients is accompanied by increased sodium uptake via sodium–glucose cotransporters (SGLT). This causes a decrease in distal sodium supply to the macula densa and an increase in renin and angiotensin II (Ang II) release. Ang II has a selective vasoconstrictor effect on the efferent arteriole, which not only raises intraglomerular pressure and filtration fraction, but also reduces tubulointerstitial oxygen delivery. In addition, Ang II exerts a positive effect on proximal sodium reabsorption, thereby increasing tubular cell oxygen consumption.

The diabetic kidney is further characterized by mitochondrial dysfunction, resulting in decreased ATP generation, which precedes the histological and biochemical features of chronic kidney disease (CKD). As consequence, the state of hypoxia in the kidney of patients with DM leads to glomerular and tubulointerstitial injury, imbalance in the synthesis and degradation of the extracellular matrix, and renal fibrosis [19,20], which in turn may exacerbate hypoxia due to oxygen transport restriction. Despite advances in the pathophysiologic understanding of DKD, clinical translation remains limited, mainly due to the lack of noninvasive methods to assess renal microcirculation, oxygenation, inflammation, and fibrosis. Renal biopsy remains the gold-standard method for analyzing renal microstructure; however, its use in DKD is often reserved for atypical clinical manifestations. Novel renal MRI tools are increasingly available to assess renal perfusion, oxygenation, and fibrosis noninvasively, offering a great potential to shed light onto the pathogenesis of DKD.

### 2.1. Perfusion

Physiologically, renal perfusion provides the pressure necessary for glomerular filtration to occur, as well as supplying nutrients and oxygen to meet the demands of renal metabolism. In the early course of DKD, due to a state of hyperfiltration, this balance is particularly overdrawn [2]. Hyperfiltration is secondary to decreased afferent arteriolar resistance mediated by vascular factors such as nitric oxide, angiotensin (1–7), and hyperinsulinemia, as well as increased efferent arteriolar resistance due to angiotensin-II, endothelin-1, or reactive oxygen species [21]. All these factors generate a renal microcirculatory disturbance that often goes unnoticed but contributes to structural renal injury [10]. In fact, there may be a significant loss of GFR reserve despite a rate greater than 100 mL/min/1.73 m^2^ and the absence of severe albuminuria (urinary albumin/creatinine ratio above 300 mg/g) [21].

The evaluation of renal microcirculation remains a challenge, due to the difficulty of its direct measurement, the considerable consumption of time and technical personnel, and the risk it may involve for the patient, depending on the technique employed. Traditional methods include dynamic contrast-enhanced MRI, which uses contrast media in combination with MRI to allow the estimation of renal perfusion; however, the use of exogenous contrast media presents the possibility of contrast nephrotoxicity and, specifically in the case of gadolinium-based contrast agents, the risk of developing nephrogenic systemic fibrosis in patients with CKD, which consists of a severe fibrotic skin disorder due to sequestration of its free ionic form (Gd^3+^) in the skin tissue, highly toxic in humans and without a currently effective treatment [22,23]. Another method consists of doppler sonography, which is able to detect alterations of renal arteries/veins and to provide an indirect measure of intrarenal vascularization through resistance indices; however, it does not allow direct quantification of renal perfusion. New developments in the field of sonography promise interesting and clinically useful results; therefore, it will be interesting to see their development in the coming years [24]. On the other hand, more precise methods such as endogenous clearance of para-aminohippuric acid (PAH) or radionuclide scintigraphy require invasive techniques, as well as significant human and time resources, without ruling out the possibility of anaphylaxis reactions. Hence, there is great importance of developing a tool that quantifies renal tissue perfusion in a more precise and less invasive manner.

### 2.2. Oxygenation

Renal oxygenation is based on a balance between oxygen supply and consumption. To comprehend this balance, it is helpful to remember the anatomy of the renal vasculature. Renal circulation depends on the renal artery, which branches into interlobular arteries extending along the renal cortex. At the corticomedullary junction, these arteries become arcuate arteries, which branch into radial cortical arteries, from which afferent arterioles arise supplying blood to the glomeruli. These glomeruli are drained by the efferent arterioles, which in their juxtamedullary portion supply the renal medulla, through the capillaries of the vasa recta [2]. The juxtamedullary glomeruli comprise only 10% of all renal glomeruli [25]. Unlike the renal cortex, no arteries penetrate the renal medulla. For these reasons, all blood flow to the kidney (25% of cardiac output) passes through the renal cortex, but only about 10% enters the renal medulla [2], making it particularly vulnerable to hypoxia. The blood flow of the outer medulla corresponds to 40% of the total blood flow of the renal cortex, whereas the inner medulla receives 10% of what the entire renal cortex receives. These characteristics differentiate the mechanisms of regulation and oxygenation of the RBF at the cortical level from those at the medullary level. In addition, renal medullary oxygenation is limited by the countercurrent exchange of the descending and ascending vasa recta [25], as well as by a lower medullary hematocrit compared with arterial blood [26]. Thus, the partial pressure of oxygen at the renal level, which decreases from 50 mmHg in the cortex to 10 mmHg in the inner medulla [27], depends on the local blood flow, the rate of oxygen metabolism, the distance of the local tissue to the nearest blood supply, the oxygen permeability of the surrounding tissue, and the oxygen saturation of the hemoglobin reaching the renal capillary beds. These factors result in a complex and multifactorial process, in which various regulators are involved. For instance, the degree of medullary hypoxia depends on the contractile action of pericytes, which provide medullary blood flow, as well as autocrine/paracrine agents, such as nitric oxide, adenosine, or ATP, which may influence the function of these pericytes [2]. Under conditions such as diabetes, where there is oxidative stress and impaired nitric oxide function, the efficiency of cellular oxygen utilization is reduced.

As a result, small variations in blood flow and/or local oxygenation can lead to tissue hypoxia, as renal oxygenation depends on several tightly regulated mechanisms. High oxygen demand is due to tubular sodium reabsorption, which consumes more than 80% of the oxygen supplied to the kidney [28]. In addition to renal metabolism, however, oxygen consumption varies according to GFR and RBF. Consequently, the renal balance between oxygen supply (RBF, hemoglobin, blood pH, and oxygen saturation) and oxygen consumption (tissue metabolism, filtration fraction, and active tubular electrolyte transport) [29] is very important for kidney function.

Renal hypoxia is known to play a major role in the progression of DKD [30,31]. Sustained hyperglycemia results in increased kidney oxygen consumption, partly due to mitochondrial dysfunction and reduced electrolyte transport efficiency [29]. Tissue hypoxia is recognized as a unifying pathway to CKD in these subjects. In particular, oxidative stress has been identified mainly in the renal medullary zone [32]. Consequently, assessing renal oxygenation is also highly relevant to clinical practice.

### 2.3. Microstructure

Renal biopsy is currently the most valuable tool to assess the renal microstructure. However, it is an invasive procedure which carries the risk of side-effects and sampling bias, as it represents less than 0.002% of the whole kidney. This fact is relevant in DKD because the early injury is patchy and with a wide variety of histological features [16].

Hyperglycemia stimulates type IV collagen synthesis, mediated by transforming growth factor-beta (TGF-β) [33], whose deleterious fibrotic effect is observed in advanced phases of DKD, at both the glomerular and the tubulointerstitial levels. Local inflammation phenomena are involved in this process, with recruitment of inflammatory cells [34], imbalance in the synthesis/degradation of the extracellular matrix [35], and vascular dysfunction [36]. Renal fibrosis is a hallmark feature of progressive DKD, being also related to the decline in kidney function. However, there are currently no validated biomarkers for quantitative and spatial determination of renal fibrosis.

## 3. Magnetic Resonance Imaging and Clinical Utility

**Figure 1:**
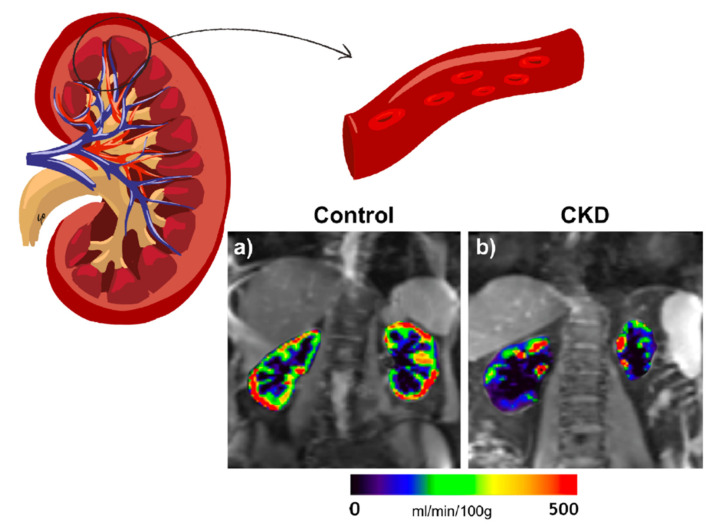
Representative RBF maps in a healthy volunteer (age 66 years) (**a**) and an individual with diabetes and CKD (age 76 years, eGFR 46 mL/min/1.73 m^2^) (**b**). The color bar applies for both control and CKD. RBF maps of the kidneys are overlaid on the reference images (M0).

**Figure 2:**
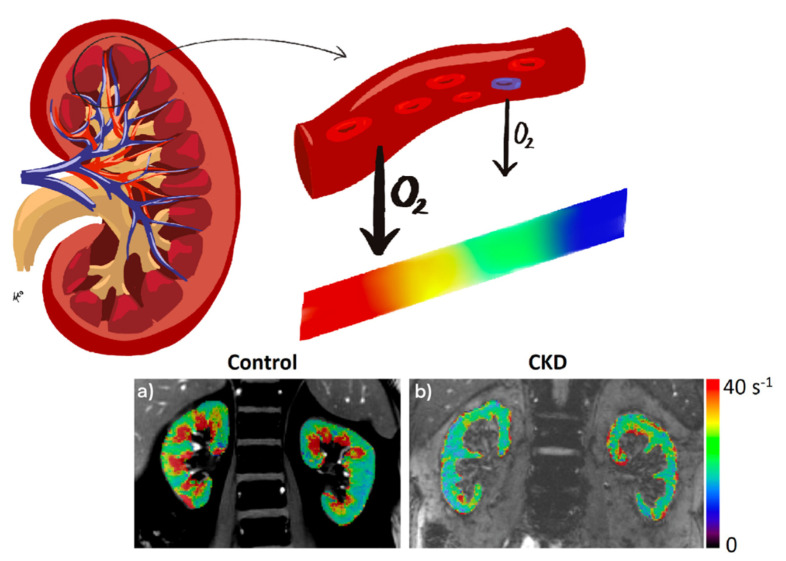
Representative R2* parametric maps in a healthy volunteer (age 62 years) (**a**) and an individual with type 1 diabetes and CKD (age 63 years, eGFR 34.7 mL/min/1.73 m^2^) (**b**). The color bar applies for both control and CKD. R2* maps of the kidneys are overlaid on the anatomic MR images (short TE images from BOLD MRI acquisition).

**Figure 3:**
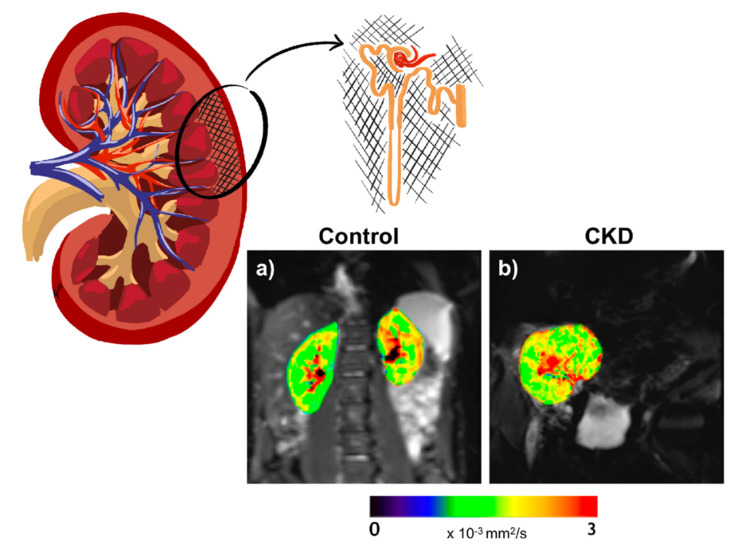
Representative ADC maps in a healthy volunteer (age 28 years) (**a**) and an individual with kidney transplant and CKD (age 69 years, eGFR 62 mL/min/1.73 m^2^) (**b**). The color bar applies for both control and CKD. ADC maps of the kidneys are overlaid on the diffusion image with gradient b = 0.

Since the early 1990s, MRI has gradually been applied for a deeper understanding of renal pathogenesis caused by diabetes [37,38]. Subsequently, the progress in these kinds of techniques has been remarkable [39,40]. Renal magnetic resonance techniques are currently in vogue as they provide noninvasive information on renal volume, function, metabolism, perfusion, oxygenation, and microstructural alterations, without the need for exogenous contrast media (Figure 1, Figure 2 and Figure 3). In the last 6 years, various international groups have emerged, composed of medical imaging experts, physiologists, physicists, engineers, radiologists, and nephrologists, among other professionals, with the aim of implementing the clinical application of these tools [41,42]. Due to its heterogeneous nature and high economic and health impact, DKD is a pathology with special appeal for the implementation of these bioimaging procedures. A recent study in a CKD population (27% with diabetes) demonstrated the usefulness of multiparametric MRI in revealing functional and histological renal alterations [43].

### 3.1. ASL

Arterial spin labeling (ASL) MRI applies a radiofrequency pulse to the circulating aortic blood on its course toward the renal arteries, which generates a magnetization inversion effect resulting in magnetically labeled arterial blood water that can be utilized as an endogenous tracer. Thus, a perfusion-weighted image results from the subtraction of a labeled image from a control (i.e., nonlabeled) image, where the ASL signal is directly proportional to the RBF, expressed in mL/min/100 g of tissue [44] (Figure 1). The ability of ASL to measure RBF has been confirmed in comparison with gold-standard methods [45,46]. Its reproducibility and correlation with conventional kidney function measurement techniques have also been established [47,48]. This technique is proving to be one of the most relevant in the study of DKD thanks to its ability to detect small changes in the renal microvasculature, in a pathology in which microvascular involvement plays a crucial role in both the onset and the progression of the disease. As discussed below, this makes it a promising tool for the development of new therapeutic alternatives and the monitoring of therapy already established, as well as the possibility of detecting early alterations and assessing disease progression [49,50].

A previous ASL study in diabetic patients [48] observed a 28% reduction in renal cortical perfusion in comparison with healthy subjects. A smaller but still significant difference in RBF was found between the healthy controls and a subgroup of diabetic patients in spite of not showing differences in kidney function according to the methods traditionally used. In addition, when patients were grouped according to the category of DKD, lower perfusion was observed as kidney failure progressed. This result was supported by a significant correlation between RBF and GFR, estimated by creatinine and cystatin-C. Other studies have shown similar results. Recently, Brown et al. analyzed in a pilot study the ability of ASL to provide information on fibrotic changes occurring during DKD [51]. To do so, they studied diabetic subjects from early to advanced categories of kidney disease and compared them with healthy volunteers using magnetic resonance elastography and ASL. The findings showed positive correlations among shear stiffness, cortical perfusion, and surrogate filtration fraction (eGFR/ASL). All three parameters decreased as DKD progressed. This study provides further evidence of ASL capacity to detect decreased RBF as DKD progresses. In combination with eGFR, it allowed estimation of the surrogate filtration fraction, which appeared to correlate with increased fibrosis, demonstrated by kidney biopsy. In another recent study, diabetic patients with a mean estimated GFR of 51 mL/min/1.73 m^2^ were analyzed for cortical perfusion, diffusion, and oxygenation. Prasad et al. not only observed a significant decrease in renal cortical perfusion in individuals with diabetes and moderately reduced GFR, compared to healthy subjects, but also an association of cortical perfusion and medullary response to furosemide with annual loss of kidney function [52]. As such, ASL emerges as a marker of incipient and subclinical damage, as well as a plausible predictor of DKD progression.

### 3.2. BOLD

Blood oxygen level-dependent (BOLD) contrast is a noninvasive method to evaluate renal tissue oxygenation on the basis of the paramagnetic properties of deoxyhemoglobin. This paramagnetic capacity of deoxyhemoglobin differs from that of the surrounding tissues generating susceptibility-induced magnetic field gradients that alter the transverse relaxation decay rate R2* (R2* = 1/T2*). The decay rate increases at higher deoxyhemoglobin concentration, which is inversely proportional to tissue oxygenation. Thus, an increased relaxation rate (R2*) denotes a higher tissue deoxyhemoglobin level and, therefore, lower oxygenation (Figure 2). Since the partial pressure of oxygen in blood is considered to be in close equilibrium with the underlying tissue, changes estimated by BOLD can be interpreted as changes in the partial pressure of tissue oxygen. This technique has been validated in various studies [53,54,55], but these validations have only been performed in animal models, because the gold-standard method for comparison is by direct measurement of tissue pO_2_, which requires micro-electrodes located in each kidney.

In regard to clinical application of BOLD-MRI in DKD, a recent and interesting study demonstrated relative renal hypoxia in T1D adolescents, associated with increased albuminuria, renal plasma flow, fat mass, and insulin resistance [56]. This finding paves the way for further investigation into the role of renal hypoxia in the onset of DKD. Moreover, a recent study in CKD subjects, 42% of whom were diabetic patients, showed that BOLD-MRI is able to predict long-term CKD progression [57]. This was previously also reported by Pruijm et al. [58], who showed using BOLD-MRI, in CKD patients (22% with diabetes) followed for 3 years, an association between renal tissue hypoxia and kidney function decline. In this prospective study, they concluded that the annual decline in GFR is more accelerated when the oxygenation of the renal cortex is more diminished. Additionally, those patients with lower oxygenation in the outermost areas of the cortex were three times more likely to develop an adverse kidney outcome. A recent analysis [52] of medulla-to-cortex ratio in R2* showed a significant difference between diabetic patients with moderately reduced GFR and a control group. This was also observed in earlier DKD [59]. Previously, other studies analyzed both renal cortical and medullary oxygenation in diabetic subjects, from early to advanced categories of CKD, observing a positive correlation between medullary oxygenation and GFR and a negative correlation between cortical oxygenation and GFR [60]. Former reports have shown that hypoxic medullary changes can be detected by BOLD-MRI in early DKD [60,61,62], which agrees with an increased oxygen consumption in the medulla due to oxidative stress during diabetes [32], and they do not seem to depend solely on alterations in renal blood flow [62]. The effect on renal tissue hypoxia secondary to nitric oxide inhibition has also been evaluated by BOLD-MRI [63,64]. Renal oxygenation was also investigated by BOLD-MRI in diabetic patients undergoing water overload to determine whether water diuresis would enhance renal tissue oxygenation, with no improvement observed with the intervention [65,66].

Nevertheless, other investigations have presented contradictory results when studying DKD. Wang et al. found lower medullary R2* values suggesting higher oxygenation in DKD patients [67]. The authors ascribed the discrepancies to the fact that their patients had overt DKD, which, in view of the fall in GFR, presupposes a decrease in tubular sodium reabsorption and, therefore, a decrease in oxygen consumption, conversely to what is observed in situations of hyperfiltration in early DKD. A previous study [68] in CKD patients observed a good correlation between BOLD T2* values and GFR. However, upon subanalysis in patients with diabetic nephropathy (30% of all CKD patients), no significant correlation was observed. Thus, the R2* increase in humans with diabetes and/or CKD is still unclear with regard to whether it is related to a concomitant decrease in RBF; therefore, future studies should be conducted to clarify the role of BOLD in people with these pathologies.

One aspect to keep in mind concerning the BOLD-MRI mechanism is that it is based on the assumption that blood and tissue oxygenation are at equilibrium. Thus, external factors such as fibrosis, anemia, or ischemia, which could restrict oxygen exchange between renal microvasculature and interstitium, could have significant effects on the results. These factors are of relevance in diabetic patients due to a higher prevalence of renal interstitial fibrosis, likely related to the described macrophage infiltration [3], anemia, and associated vascular disease in this profile of patients [5]. Additionally, hydration status and salt intake have also been shown to influence renal oxygenation. A significant decrease in medullary R2* was observed after oral water load [69] and high-salt intake [70].

Another reason for these discrepancies is the lack of consensus on how to analyze BOLD-MRI images. For this reason, a number of studies have emerged to improve and homogenize the acquisition and analysis protocols of the technique [55]. In fact, a group of experts created an interesting consensus statement seeking standardization of measurement protocols by giving technical recommendations on imaging parameters according to some patients’ situations, as well as patient preparation prior to the examination [71].

### 3.3. DWI/DTI

Diffusion-weighted imaging MRI (DWI-MRI) is a technique which has emerged as a promising method for assessing renal microstructure. By applying diffusion gradients, it generates image contrast that is sensitive to water motion in the kidney tissue. Accordingly, it is a tool to probe the renal microstructure, able to estimate the presence of renal fibrosis and/or cellular infiltration (Figure 3). Thus, a lower diffusion capacity may indicate greater fibrosis, as a fibrotic renal interstitium decreases the Brownian motion of the water molecules by collision. The apparent diffusion coefficient (ADC) consists of the overall measure of water diffusion and microcirculation in the tissue. ADC can be measured by DWI by acquiring images with different diffusion weighting factors (or b-factors). However, as molecular diffusion occurs in a three-dimensional space, it is not equal in all directions. For this reason, although ADC measures global diffusion, it is not able to describe the directionality of the movement. Diffusion tensor imaging MRI (DTI-MRI) measures the diffusion signal in a greater number of directions, allowing an analysis of the spatial dependence of diffusion and, thus, the tissue microarchitecture, through the quantification of tissue anisotropy (fractional anisotropy) (0% = complete isotropy, 100% = complete anisotropy) [72]. Fractional anisotropy (FA) gives information on the microstructural orientation as a percentage of spatially oriented diffusion signal. Hence, DTI-MRI evaluates the directionality of water mobility (indicated by FA), as well as its magnitude (measured by ADC). Under healthy circumstances, there is a greater diffusion of water in the cortical zone of the kidney compared with the medullary region, but this relationship could be inverted in patients with DKD; therefore, a greater diffusion of water estimated by ADC may be seen at the medullary zone than in the cortical area. This inversion in the corticomedullary differences correlates with greater renal fibrosis. Other physiological situations may also alter the DWI signal and induce changes in ADC (renal perfusion, tubular and glomerular flow, or water handling) [73,74]. Intravoxel incoherent motion diffusion-weighted imaging (IVIM-DWI) is a method within DWI which acquires images with multiple b-values and uses a biexponential pattern to extract quantitative information, minimizing the influence of blood and tubular fluid flows. A recent study by IVIM-DWI identified restricted water diffusion in the renal tissue of T2D patients with a relatively intact kidney function [75].

Several studies have shown the benefit of DWI-MRI in the study of diabetes. Decreased ADC has been observed in renal parenchyma of T2D subjects in comparison with healthy volunteers. In the same study, ADC was negatively correlated with fasting blood glucose [59]. Lu et al. reported lower medullary FA and ADC in diabetics subjects compared to controls [76]. The medullary FA was also correlated with kidney function [76,77], suggesting that DTI-MRI allows evaluating renal fibrosis in DKD. These findings were recently confirmed in patients with mild DKD, including the ability to detect renal lipid deposition [78], and in diabetic murine models, finding association with histopathological changes of interstitial fibrosis and glomerulosclerosis [79,80,81]. However, a recent study performed in CKD patients who underwent renal biopsy of either native kidney (22% of whom had DKD confirmed by renal biopsy) or allograft kidney (none with DKD in the graft) showed an interesting result, suggesting that the corticomedullary ADC difference correlates better with interstitial fibrosis than absolute cortical or medullary ADC values [82]. Hence, it would allow a better identification with greater specificity of those patients with interstitial fibrosis. Inoue et al. analyzed almost 50 diabetic subjects with all categories of GFR and albuminuria, compared with 76 healthy controls, concluding that ADC allows accurately detecting tubulointerstitial alterations at the renal cortex level [68]. Further studies demonstrated a significant correlation between ADC and GFR [77,83]. In turn, Çakmak et al. observed, in 78 T2D subjects with DKD aged between 26 and 70 years, that ADC showed significant correlation with clinical categories of DKD [84]. However, Prasad did not observe significant differences between diabetic subjects with moderately reduced GFR and healthy volunteers or an association with eGFR, but ADC was indeed associated with renal perfusion [52]. This result may be due to moderately reduced GFR, as, in severely reduced GFR, lower ADC values were demonstrated in a diabetic population compared with healthy controls [85]. Another study, also in T2D but with low–moderate CKD risk (G1, A1–A2), showed that the combination of ADC with FA values is able to yield information about renal involvement even in early DKD [86]. However, FA seems to be more sensitive to detect these alterations [77]. More recently, another study in T2D concluded that both parameters (ADC and FA) are able to differentiate between healthy and diabetic volunteers, as well as predict the presence of severe albuminuria and correlate with kidney injury biomarkers, such as serum creatinine, urinary TGF-β1, and *N*-acetyl-beta-d-glucosaminidase [87].

A third available tool is diffusion kurtosis imaging (DKI). It evaluates the non-Gaussian motion of water. This method avoids the artefact caused by structural barriers at the level of the renal medulla which affect the diffusion of water molecules that do not follow a Gaussian distribution. In the presence of early structural alterations in DKD, this tool shows promise for assessing pathological changes in the diabetic kidney (thickening of the glomerular basement membrane, glomerulosclerosis, and tubulointerstitial alteration) from early categories of disease [88].

## 4. Drug Therapies Evaluation

A potential area of application of MRI biomarkers is the selection of personalized treatments in patients with diabetes both with and without established DKD on the basis of a more specific knowledge of the mechanisms affecting each individual. It would also be very useful in the monitoring of possible nephrotoxic effects of new drugs under development.

In the last years, SGLT2 inhibitors have shown an important efficacy in the prevention of progressive DKD [89]. During hyperglycemia, there is an increase in glucose delivery to the proximal convoluted tubule (PCT), which results in increased tubular glucose uptake and increased sodium reabsorption, through the sodium–glucose cotransporter-2 (SGLT2). This generates a diminution in sodium delivery to the distal tubule, sensed by the macula densa as a reduction in effective circulating volume. Through tubuloglomerular feedback, this situation generates vasodilatation of the afferent arteriole and vasoconstriction of the efferent arteriole. These hemodynamic changes lead to intraglomerular hypertension and hyperfiltration, causing podocyte stress [15]. In the last few decades, the only treatment considered the cornerstone in the prevention and management of DKD has consisted of RAS inhibitors. RAS inhibition reverses vasoconstriction of the efferent arteriole, reducing glomerular afterload. Recently, SGLT2 cotransporter inhibitors (SGLT2i) have emerged as an attractive option due to their effect on the restoration of tubuloglomerular feedback and the reversal of hemodynamic changes [18], as well as for improving the metabolic profile of patients by increasing renal excretion of glucose [90]. Despite this, few tools exist for noninvasive, real-time assessment of renal pharmacokinetics and pharmacodynamics of new therapies, such as SGLT2i or glucagon-like peptide 1 receptor (GLP-1) agonists.

In this line, novel MRI techniques are of particular interest because of their noninvasive design and proven clinical feasibility. Preliminary results have shown that they allow the assessment of renal microperfusion, as well as edema, tissue oxygenation, and renal fibrosis. Several studies have demonstrated the validity of these tools in pharmacological analysis and compartmental individualization of the kidney, providing information on the function of each kidney separately and its compartments (cortex and medulla) [45,55,83,91,92]. For instance, a possible clinical application in pharmacology would be the validation of the Gomez equations for estimating the renal hemodynamic profile [93], replacing the need to use PAH clearance by quantification of RBF by ASL-MRI.

Previous reports showed in patients using RAS blockers an increase in RBF detected by ASL-MRI [45,91]. Ritt et al. demonstrated a significant positive correlation between the change in renal perfusion after the use of RAS blockers (telmisartan), quantified by ASL-MRI, and the change exerted with the same drug after estimation by PAH clearance [45]. ASL-MRI revealed that RAS inhibition with losartan was able to reverse the detrimental cortical perfusion present in some kidney allograft recipients [91].

In several studies, BOLD-MRI showed the response in renal medullary and cortical oxygenation to certain selective diuretics [60,94,95]. As it is known, furosemide causes an inhibition of active transport in the ascending limb of the loop of Henle, which leads to a lower oxygen consumption and, therefore, medullary oxygenation improvement [53]. This effect was quantified by BOLD-MRI in murine models [94,96] and in combination with acetazolamide [55,95]. Analyzing CKD subjects (33% with diabetes), the oxygenation response to furosemide was blunted in the medulla, as denoted by a flatter R2* slope [55]. BOLD-MRI has also been useful to evaluate the nephrotoxic effect of iodinated contrast agents in experimental DKD models [97].

DWI-MRI, as a potential biomarker of fibrosis, demonstrated the renoprotective effect of miR-29a in DKD [92].

## 5. Conclusions

There is still room for integration of magnetic resonance techniques in clinical practice. A multicenter clinical trial currently underway (iBEAt study―NCT03716401) will be crucial for the development of prognostic imaging biomarkers for DKD. The study is part of the BEAt-DKD project and aims to examine the gradual changes in imaging biomarkers as kidney failure progresses in the same subject, through long-term follow-up (3 years) of a large number of patients (*n* = 500) with T2D and various categories and features of DKD. It also seeks to validate imaging biomarkers in comparison with histological data and against ^15^O water positron-emission tomography [98].

Several MRI techniques are currently being developed that would be of interest in the assessment of diabetes; nevertheless, technical validation is still required. Among them, quantitative magnetization transfer analyzes the spin exchange between proton clusters in different environments to assess the macromolecular content of the tissue. Quantitative magnetization transfer has been used to explore and quantify renal fibrosis in DKD [99]. Furthermore, dynamic nuclear polarization MRI technique was recently used to study the pathophysiological role of renal hypoxia in diabetes [100]. However, further studies are still needed to validate these MRI techniques.

The MRI tools described here have shown an important development in the last 10 years; however, work is yet to be done in the standardization of protocols to reduce the outcome differences across working groups and facilitate clinical translation, in addition to continuing stimulating research to improve the predictive power of these tools [101]. Looking ahead, an important concept to promote is the possibility of combining different bioimaging methods in order to acquire a more comprehensive view of renal pathophysiology, in highly prevalent diseases such as DKD, with the aim of improving predictions of CKD progression and gaining a better understanding of therapeutic tools. A possible pitfall in the use of these procedures is the presence of situations that may affect the results, such as the anemia, hypoxia, or fibrosis while using BOLD-MRI or vascular alterations in ASL-MRI. In addition, it is important to emphasize that MRI imaging techniques enable estimation of processes that are common to various diseases; therefore, their role is not intended to distinguish between diverse entities when the differential diagnosis is based on molecular or ultrastructural aspects, such as the presence of IgA or subepithelial deposits and/or podocyte foot process effacement. For this, renal biopsy plays a crucial role due to the information it provides. Furthermore, we must be aware that the measurement of biomarkers by MRI presents a high economic cost compared to urinary and blood biomarkers. For this reason, it is necessary to carry out cost-effective studies to determine whether the elevated initial cost justifies an economic benefit to the healthcare system. Improvements in pharmacological knowledge may orient toward a more personalized DKD patient management, as well as find indicators that allow us to prevent the onset or progression of DKD.
